# Perceived Nursing Interruptions During Medication Administration: Development and Validation of a Questionnaire Among Chinese Nurses

**DOI:** 10.1155/jonm/5881570

**Published:** 2026-09-07

**Authors:** Xiaoqian Dong, Nandan Chen, Cuilan Dong, Xingxing Wang, Sha Wang, Li Yang, Gang Gan, Min Liu, Jianfei Xie

**Affiliations:** ^1^ Nursing Department, Third Xiangya Hospital, Central South University, Changsha 410000, China, csu.edu.cn; ^2^ Xiangya School of Nursing, Central South University, Changsha 410031, China, csu.edu.cn; ^3^ Nursing Department, Hunan Children’s Hospital, Changsha 410021, China, hnetyy.net; ^4^ Clinical Nursing Safety Management Research Center, Central South University, Changsha 410031, China, csu.edu.cn

**Keywords:** medication administration, nurses, nursing interruptions, questionnaire, reliability, validity

## Abstract

**Background:**

Nursing interruptions during medication administration occur frequently, often leading to medication errors and workflow delays that threaten patient safety and reduce nurses’ work efficiency. Current research has relied mainly on observation or interviews, which are labor‐intensive, time‐consuming, and prone to substantial variability. Practical, standardized instruments are needed to assess nursing interruptions during medication administration (NIMA).

**Methods:**

This cross‐sectional descriptive study developed and validated the questionnaire for measuring nursing interruptions during medication administration (Q‐NIMA) through a multistep, theory‐driven process based on the Systems Engineering Initiative for Patient Safety model. Items were generated from literature and expert input, refined by two Delphi rounds with 12 experts, and piloted with 30 nurses. Item analysis was conducted for comprehensive evaluation, and validity evidence was gathered based on test content, internal structure, and relations to other variables. The sample was split for exploratory factor analysis (EFA) using FACTOR software (based on polychoric correlations and ULS extraction) and confirmatory factor analysis (CFA) using AMOS. Reliability was assessed using internal consistency (Cronbach’s *α* and ORION coefficients) and stability (test‐retest reliability).

**Results:**

We selected 478 nurses from a tertiary hospital in Hunan Province from July to August 2025. The final questionnaire comprised 25 items across five dimensions. Item analysis indicated that the questionnaire had strong discriminant validity. The content validity indexes were 0.944 at scale level and 0.833–1.000 at item level. EFA extracted five factors explaining 74.25% of the total variance. CFA demonstrated good model fit (*X* ^2^/df = 2.455, CFI = 0.942, TLI = 0.933, RMSEA = 0.078). Criterion validity showed a significant positive correlation (*ρ* = 0.310, *p* < 0.001) between the Q‐NIMA and the NASA‐TLX. The scale demonstrated excellent reliability: the total Cronbach’s *α* was 0.973, split‐half reliability was 0.951, and test‐retest reliability (ICC) was 0.992. The five dimensions were labeled: nurse professional competence, nurse psychophysical state, patient‐related interruptions, workplace colleague factors, and work system factors.

**Conclusion:**

The Q‐NIMA demonstrates robust psychometric properties, including high reliability, validity, and stability. Furthermore, it serves as a valuable tool to evaluate how nurses perceive nursing interruptions during medication administration. By aligning items with medication tasks and work‐system elements, the Q‐NIMA moves beyond event counting to support risk profiling and targeted improvement of medication administration.

## 1. Introduction

Nursing interruptions (NI) are events that interrupt or delay ongoing nursing procedures and divert nurses’ attention from the delivery of care for patients under a specified time, role, and environment [1, 2]. NI frequently occur among nurses in clinical settings, including emergency departments [3], pediatrics [4], and intensive care unit (ICU) [5], among others. Among various nursing tasks, NI during the medication administration (NIMA) are particularly prevalent [6]. Most studies conventionally define NIMA as interruptions encountered by nurses while implementing medication orders, including documentation, transcription, verification, preparation, administration, and prompting or supervising patients to take medications [7]. For example, a prior study reported that the rate of NIMA among pediatric nurses reached 94.51% [4]. Across a 270‐h observation in a Chinese hospital, nurses experienced 3424 NIMA events, corresponding to 12.68 interruptions per hour and accounting for 9.87% of the observation time [8].

Although some interruptions may be necessary or beneficial depending on context, evidence links NIMA to adverse patient and workflow outcomes [9], including medication errors [10], delays in care [11], missed nursing care [12], reduced work quality [13], and increased workload [12]. NIMA are also associated with poorer occupational outcomes for nurses, including lower well‐being [14], reduced job satisfaction [15], and higher psychological stress [16]. In addition, interruptions coincide with degraded work environments and elevated mental workload and burnout, which may further exacerbate risk [17]. These associations underscore the need for precise, standardized measurement of NIMA to capture not only frequency and sources but also perceived severity and controllability.

Observation is advantageous because it allows immediate recording of events as they occur. Most observational studies therefore use semistructured field notes or tally sheets to capture the frequency, timing, type, and source of NI [8, 16, 18]. For example, Forsyth et al. [19] applied the work interruption tool to identify in real‐time characteristics of interruptions among emergency department nurses, including cause, priority, and location. Similarly, Healey et al. [20] developed an observational tool to record distractions and interruptions during surgery in the operating theater. Each event was classified into predefined categories and rated by the level of team involvement, and the summed ratings were used as a measure of intraoperative interference. However, observation is resource‐intensive, limits generalizability, and is susceptible to observer effects, subjectivity, and misclassification [21]. If the observer is not familiar with nursing work, data quality may be compromised, and the clinical meaning of NI can be hard to interpret, which complicates the use of observational tools [22]. Moreover, observation is poorly suited to measuring nurses’ cognitive appraisal, such as perceived severity, controllability, and disruptiveness, that mediates risk and guides behavior. In addition, mismatches between research aims and measurement methods have been reported. For example, a recent research directly linked objective interruption incidence with self‐perceived psychological stress of nurses, leading to biased interpretations [23]. These limitations underscore the need for valid and reliable self‐report instruments that complement observation by capturing appraisal and burden, thereby enabling more precise assessment of NI. Existing self‐report instruments, including the four‐item intrusion scale [24] and the workplace interruptions measure scale based on the four factor typology [25], are generic and not nursing‐specific, thus failing to reflect the task, technology, and organizational features of clinical work. To fill this gap, Yu and Lee developed the nursing work interruption scale [22], a 12‐item self‐report tool that emphasizes human and environmental sources and has since been translated into Persian [26] and Chinese [27]. However, it does not target the medication administration context, by focusing only on human and environmental domains, and overlooks task, tools and technology, and organizational determinants of NIMA. Consequently, the scale provided limited guidance for managers, as it failed to identify aspects of the work system should be prioritized for improvement.

NIMA arises from multiple work‐system components. Guided by the Systems Engineering Initiative for Patient Safety (SEIPS) [28], we organize determinants across tasks, person, physical environment, tools and technology, and organization. Task factors include the criticality and interruptibility of multistep processes such as preparation and administration, with intravenous medications showing about one interruption per eight administrations [29]. Person factors include nurses’ self‐efficacy and stress, which increase attentional lapses and are linked to medication errors [30], and external actors who initiate interruptions, including patients, family members, physicians, and colleagues [8]. Physical environment factors include noise, crowding, and suboptimal lighting in medication rooms [31]. Tools and technology factors include device alarms, system latency, and mobile phone alerts that are common in emergency and intensive care units [32]. Organizational factors include staffing levels, scheduling, and role assignment [33].

Chinese nurses face persistent system level constraints, including staffing shortages [34] and excessive workload [35], which undermine safe, high quality care and increase the likelihood of interruptions. At present, there is no Chinese instrument that specifically measures NIMA. The absence of such a tool prevents accurate assessment of exposure and burden and limits the design and evaluation of targeted interventions. To address this gap, this study aims to develop and validate a standardized self‐report questionnaire for measuring NIMA (Q‐NIMA), which supports nursing management practice by identifying interruption patterns, guiding interventions, and evaluating outcomes.

## 2. Methods

### 2.1. Design

An observational cross‐sectional study was conducted in two phases. In the first phase, we developed the items comprising the Q‐NIMA and conducted a pilot study to test its item clarity. In the second phase, we conducted a final validation study in which we assessed the validity and reliability of the Q‐NIMA.

### 2.2. Setting and Participants

From July to August 2025, we conducted a cross‐sectional survey using convenience sampling to investigate clinical nurses in a tertiary university‐affiliated teaching hospital in China. The hospital has 2200 beds across 62 nursing units, employing 3447 staff members, including 1459 registered nurses. All nurses receive annual training on medication administration, medication errors, and patient safety provided by the nursing department. This study was conducted in accordance with the Strengthening the Reporting of Observational Studies in Epidemiology (STROBE) guidelines (see Supporting Table 1: STROBE statement). A sample of 30 participants who did not participate in the final validation study was recruited for the pilot study. A sample of 500 participants was recruited for the final validation study. The inclusion criteria were (a) holding a registered nurse license, (b) full‐time employment with at least 1 year of clinical experience, and (c) working in the current department for no less than 3 months. Exclusion criteria included nurses who were on personal leave, maternity leave, sick leave, study leave, or rotation; visiting nurses; and those whose work did not involve medication administration (e.g., disinfection supply center staff).

### 2.3. Sample Size

The initial sample size determination was guided by traditional recommendations for scale development, aiming for a minimum of 200 cases for factor analysis [36]. Methodological guidelines further recommend a sample of 150–200 cases for exploratory factor analysis (EFA) and ≥ 200 cases for confirmatory factor analysis (CFA). However, recent methodological guidelines emphasize that adequate sample size is not solely dependent on the number of items but is contingent on data characteristics, specifically item communalities and factor overdetermination [37, 38]. Based on these considerations, we distributed 500 questionnaires and collected 478 valid responses (95.6% response rate). The sample was split into two independent subsets: 239 cases collected in July were used for item analysis, reliability testing, and EFA, while the 239 cases collected in August were allocated to CFA. This sample size ensured sufficient statistical power to support both exploratory and confirmatory analyses.

### 2.4. Development and Pilot Study of the Q‐NIMA

#### 2.4.1. Establishment of the Initial Item Pool

This study was informed by the SEIPS model [28], which conceptualizes the work system in five components, person, tasks, tools and technology, physical environment, and organization. The previously established risk evaluation index system for NIMA served as the primary source for item generation [31]. To address potential gaps regarding the “person” component specifically related to nurses, we conducted a focused literature review and drafted additional items assessing nurse professional competence (NPC) and psychophysical state. Following consolidation, refinement, and deduplication, the initial item pool was established.

To better operationalize the SEIPS framework within the specific context of medication administration in China, we organized the items into five hypothesized domains. This structure was designed to distinguish the nurse’s internal attributes from external systemic factors. Specifically, the “person” component was differentiated into NPC and nurse psychophysical state (NPS) (internal factors), while the social and systemic components were categorized into patient‐related interruptions (PRI), workplace colleague factors (WCF), and broad work system factors (WSF) (integrating environmental, resource, and organizational elements). This classification allows for a more granular analysis of the sources of interruptions. The final initial pool contained 69 items.

#### 2.4.2. Consultation With Experts

Using purposive sampling, we invited 12 experts from different regions and hospitals to participate in a Delphi consultation. The inclusion criteria were as follows: professional background in nursing management, clinical nursing/medicine, nursing education, or nursing psychology; a master’s degree or above; intermediate or senior professional titles; at least 10 years of work experience; familiarity with medication nursing or questionnaire development; and willingness to provide informed consent and participate voluntarily.

Before the consultation, experts were informed of the study’s objectives and significance, and their consent was obtained. The Delphi inquiry form was distributed via email, consisting of three parts: (1) terminology and an introduction to the research background, purpose, and content of the study; (2) expert evaluation of the relevance, importance, and feasibility of questionnaire items related to NIMA; and (3) basic information such as gender, age, job title, and field of expertise. Experts were invited to provide comments on the professional content, clarity, and formatting of the questionnaire. Item selection criteria were set as mean importance score > 3.50, coefficient of variation < 0.25, and total score ratio > 20% [31, 39]. Based on expert feedback, the research team (comprising academic supervisors, clinical nursing experts, nursing administrators, graduate students, and clinical nurses) held group discussions and revised the item pool by adding, deleting, or modifying items to improve the representativeness and clinical applicability of the preliminary questionnaire.

#### 2.4.3. Pilot Survey

We conducted a preliminary survey in July 2025 by offline distributing 30 paper questionnaires (in Chinese) to nursing staff in the ICU, surgery, internal medicine, and emergency departments of a tertiary Grade A comprehensive hospital in Changsha, Hunan Province, China. The inclusion and exclusion criteria for the pilot survey were consistent with those applied in the formal survey. Nurses undergoing continuing education, rotation, or internship were excluded. Each participant was asked to rate the clarity of every item on a 0–10 scale, in addition to providing general feedback on wording and comprehensibility. All 30 questionnaires were returned, yielding a 100% valid response rate. Based on the clarity scores and comments, we made minor wording adjustments and finalized the official test version of the Q‐NIMA.

#### 2.4.4. Scale Response Format

Previous NI instruments typically adopt frequency‐based response options with fixed time frames, as in the workplace interruptions measure scale [25] and the nursing work interruption scale [22]. However, during our pilot work, nurses reported substantial difficulty accurately estimating the exact number of medication administrations and interruptions within defined periods, whether 1 week or 1 month. To reduce recall burden and improve feasibility, we adopted a five point Likert format with ordered frequency descriptors: never (1), occasionally (2), sometimes (3), often (4), and always (5). Nurses were instructed to judge their typical clinical shift within a recent and stable time window. This format aims to capture perceived occurrence without requiring exact counts. We will treat responses as ordered categorical data and examine category functioning and threshold ordering during psychometric analyses.

### 2.5. Data Collection

Eligible nurses were invited to participate through the Wenjuanxing online platform (https://www.wjx.cn/login.aspx). Prior to data collection, approval was obtained from the nursing department, and the head nurses of each ward assisted in identifying eligible participants and distributing the questionnaires. The survey form consisted of four sections: study information and participants’ rights; sociodemographic data; the newly developed Q‐NIMA for validation; and the NASA task load index (NASA‐TLX), a six‐item measure of perceived task load. Although the NASA‐TLX evaluates explicit mental workload, it was included to assess the criterion validity of the Q‐NIMA, given prior evidence suggesting a relationship between nursing interruption occurrence and mental workload.

### 2.6. Statistical Methods

Data analysis was performed using SPSS 26.0, AMOS 26.0, and FACTOR 12.06.07. Continuous variables are presented as means ± SD, whereas categorical variables are reported as frequencies and percentages. Following the COSMIN guidelines and the Standards for Educational and Psychological Testing (AERA, APA, and NCME, 2014), validity was conceptualized as a unitary concept supported by multiple sources of evidence. A two‐tailed *p* < 0.05 was considered statistically significant.

#### 2.6.1. Item Analysis

Item analysis was conducted to evaluate whether each entry could effectively distinguish between respondents with high and low overall scores [39]. The filtering criteria were as follows: (a) critical ratio method: items were deleted if they lacked statistically significant differences (*p* > 0.05) between the high‐scoring group (top 27%) and the low‐scoring group (bottom 27%); (b) corrected item‐total correlation: items with a correlation coefficient < 0.40 with the total score were removed; (c) internal consistency check: items were removed if the Cronbach’s *α* coefficient of the scale increased significantly after their deletion. The Q‐NIMA was interpreted as having good internal consistency if the total Cronbach’s alpha of the scale was greater than 0.7.

#### 2.6.2. Evidence Based on Test Content

Content validity was assessed to determine whether each item appropriately represented its intended construct using the content validity index (CVI). Two indices were computed: item‐level CVI (I‐CVI) and scale‐level CVI (S‐CVI). A panel of 12 experts independently rated each item’s relevance to its target dimension on a 4‐point scale (1 = not relevant, 2 = slightly relevant, 3 = relevant, 4 = highly relevant). Following established guidelines, an I‐CVI above 0.78 and an S‐CVI of 0.90 or greater were regarded as indicative of acceptable content validity [40].

#### 2.6.3. Evidence Based on Internal Structure

##### 2.6.3.1. Exploratory Factor Analysis (EFA)

EFA was conducted using FACTOR software. Before extraction, sampling adequacy was verified using the Kaiser–Meyer–Olkin (KMO) index (> 0.70) and Bartlett’s test of sphericity (*p* < 0.05) [41]. Given the ordinal nature of the Likert‐scale data, the analysis was based on the polychoric correlation matrix rather than Pearson correlations. The unweighted least squares (ULS) extraction method was applied to minimize the residuals of the factor solution. To achieve a simple and interpretable structure, Promin rotation (an oblique method) was used, acknowledging that the underlying psychological constructs are likely correlated. The number of factors to retain was determined using parallel analysis (PA) based on minimum rank factor analysis (MRFA), a method considered more robust than the traditional eigenvalue > 1 rule. Items with factor loadings below 0.40 were considered for removal.

##### 2.6.3.2. Confirmatory Factor Analysis (CFA)

CFA was performed using AMOS 26.0 to cross‐validate the factor structure obtained from EFA. Prior to the analysis, the data distribution was examined. Since the absolute values of skewness and kurtosis were within the acceptable limits (skewness < 3, kurtosis < 10), the data were treated as approximately normal, and the maximum likelihood (ML) estimation method was employed. To comprehensively evaluate the model fit, we reported a set of indices, including the Chi‐square to degrees of freedom ratio (*χ* ^2^/df), root mean square residual (RMR), root mean square error of approximation (RMSEA), incremental fit index (IFI), Tucker–Lewis index (TLI), and comparative fit index (CFI). Additionally, parsimony fit indices such as the parsimony goodness‐of‐fit index (PGFI) and parsimony normed fit index (PNFI) were also considered. Following the recommendations of Hu and Bentler [42] and strictly adhering to the criteria listed in Table 1, the model fit was deemed acceptable if *χ* ^2^/df < 3, RMR < 0.05, RMSEA < 0.08, and IFI, TLI, CFI values > 0.90. For parsimony indices (PGFI and PNFI), values > 0.50 were considered acceptable [43]. Furthermore, to ensure the robustness of the factor structure, we compared the proposed model with alternative models using the Akaike information criterion (AIC), where lower values indicate a better trade‐off between model fit and parsimony.

**TABLE 1:** Results of fitting indicators for the questionnaire model of the Q‐NIMA.

Index	*X* ^2^/df	RMR	RMSEA	IFI	TLI	CFI	PGFI	PNFI
Judgment criteria	< 3	< 0.05	< 0.08	> 0.9	> 0.9	> 0.9	> 0.5	> 0.5
After correction	2.455	0.028	0.078	0.942	0.933	0.942	0.660	0.791

#### 2.6.4. Evidence Based on Relations to Other Variables

To evaluate criterion validity, we correlated Q‐NIMA total scores with scores on the NASA‐TLX [22, 44]. We first examined distributional assumptions for the Q‐NIMA total score, and tests of normality indicated deviation from normality (Shapiro–Wilk test statistic = 0.901, df = 478, *p* < 0.05; skewness = 1.108, SE = 0.112; kurtosis = 1.066, SE = 0.223). Accordingly, we used Spearman’s rank correlation (*ρ*) to quantify the association between the two measures.

#### 2.6.5. Reliability Analysis

Internal consistency was estimated using Cronbach’s *α* coefficient, while reproducibility was examined through split‐half reliability and test‐retest reliability [39]. Following recent methodological recommendations, recognizing the limitations of Cronbach’s *α* (e.g., tau‐equivalence assumption) [45], we further evaluated the reliability of the derived factor scores using ORION coefficients (overall reliability of fully‐informative prior oblique N‐EAP scores). Computed via FACTOR software, ORION provides a more precise estimation of measurement precision for the latent factors in multidimensional scales [46]. About test‐retest reliability, the same steps were followed as in the pilot study. In addition, the stability of the scale was assessed by administering the Q‐NIMA to a subsample (*n* = 30) twice with a 3‐week interval between measurements. We analyzed the test‐retest reliability of the subscales that comprised the Q‐NIMA by calculating the intraclass correlation coefficient (ICC).

### 2.7. Ethical Considerations

Written informed consent was obtained from all participants, who were assured of their right to withdraw. All data were kept confidential. The study was approved by the Ethics Committee of Xiangya Nursing School of Central South University (No. E2022184). This study was conducted in accordance with the Declaration of Helsinki.

## 3. Results

### 3.1. Expert Inquiry Results

From May to June 2025, we selected 12 experts (four men and eight women) from Guangdong, Hunan, Shanxi, and Henan provinces, as well as Chongqing Municipality. Their ages ranged from 31 to 63 (42.33 ± 11.02) years, and their educational backgrounds included one undergraduate, five master’s, and six doctoral degrees. Work experience ranged from 4 to 44 (17.25 ± 13.74) years. There were three intermediate professionals, three deputy senior professionals, and six senior professionals. Among them, there was one clinical nurse, two nursing educators, six nursing managers, one safety manager, three medical managers. Detailed information can be found in Supporting Table 2: experts’ personal information. We conducted two rounds of expert inquiry, with expert enthusiasm coefficients of 100%; 9 experts and 7 experts gave opinions, respectively. The expert authority coefficients were 0.913 and 0.925, respectively. The Kendall harmony coefficients were 0.134 and 0.191, respectively (*p* < 0.001).

In the first round of expert inquiry, the average score of the importance of the items was 3.83–4.92, with a coefficient of variation of 0.06–0.29 and a total score ratio of 25.00%–91.67%. To improve clarity, actionability, and content validity, we conducted a systematic revision of the NIMA draft following the first round of expert consultation. Specifically, a total of 9 experts provided opinions, and after discussion, we modified or merged 17 consolidations (collapsing 46 items to 18), deleted three items, added one new item, and harmonized wording and scope across the retained items. Ultimately, 38 items in five dimensions were retained. Representative consolidations included merging six supply/consumables management items into “consumables quantity” and “materials management,” standardizing “shortage” to “insufficient preparation” to facilitate operational assessment, and merging two patient identification items into one (“I once interrupted medication administration due to patient identification problems”). Deleted items were I‐1.9 (inconsistent with current verification procedures; ceiling rate < 20%), I‐1.13 (construct measurable with existing instruments), and I‐1.16 (conceptually redundant with knowledge items, difficult to intervene on; coefficient of variation > 0.25). We added one item to strengthen the information domain (III‐3: defects in drug/consumable information such as unrecorded batch numbers or unclear expiry labels). In addition, we clarified the wording of the 18 retained items and adopted a consistent past‐tense template (e.g., “I once interrupted medication administration”) to align temporal and behavioral framing. Collectively, these adjustments reduced redundancy and respondent burden while preserving construct coverage, thereby enhancing the scale’s clinical applicability and content validity.

In the second round of expert inquiry, the average score for the importance of the items was 4.17–5.00, with a coefficient of variation of 0–0.20 and a total score ratio of 41.67%–100%. Seven experts (the remaining five experts had no revision opinion) proposed modifications. After discussion, six items were modified; however, no new items were added. Specifically, two hospital‐management items (medication safety policy and supervision/feedback) were merged into a single item: “I once interrupted medication administration because a management policy for medication‐related NI was lacking or not adhered to.” In addition, four items underwent minor wording refinements to improve clarity and maintain tense/terminology consistency; no new items were added. Consequently, five dimensions and 37 items were retained, with no alteration of the domain structure.

### 3.2. Participant Characteristics

The validation study ultimately included 478 nurses, the majority of whom were female (92.3%). The average participant age was 35.16 years (SD = 6.02). Further sociodemographic information is presented in Table 2.

**TABLE 2:** Sociodemographic characteristics of research subjects (*n* = 478).

Characteristics	Category	Overall (*n* = 478) (%)
Department type	Internal medicine	84 (17.6%)
	General surgery	162 (33.9%)
	Obstetrics and pediatrics	26 (5.4%)
	Emergency and critical care	104 (21.8%)
	Others	102 (21.3%)

Gender	Male	37 (7.7%)
	Female	441 (92.3%)

Age		35.16 ± 6.02

Educational level	Associate’s degree or below	5 (1.0%)
	Bachelor’s degree	382 (79.9%)
	Master’s degree or above	91 (19.0%)

Professional title	Junior nurse	22 (4.6%)
	Intermediate nurse	68 (14.2%)
	Senior nurse	382 (79.9%)
	The highest nurse	6 (1.3%)

Service years	≤ 5 years	77 (16.1%)
	6–10 years	78 (16.3%)
	11–15 years	99 (20.7%)
	16–20 years	165 (34.6%)
	≥ 20 years	59 (12.3%)

Shift type	Charge nurse shift	36 (7.5%)
	Primary nurse shift	350 (73.2%)
	Assistant nurse shift	11 (2.3%)
	Quality control nurse shift	12 (2.5%)
	Others	69 (14.4%)

### 3.3. Item Analysis Results

Prior to psychometric evaluation, the distributional properties of the items were examined. We observed that the response data were positively skewed, with a very low endorsement rate for the extreme category “always” (5). This floor effect is common in interruption research, as certain high‐risk interruptions are ideally rare events. To ensure robust parameter estimation and avoid sparse cells in subsequent analyses, we collapsed the response categories “often” (4) and “always” (5) into a single category. Consequently, all items were treated as four‐point ordinal variables (1 = never, 2 = occasionally, 3 = sometimes, 4 = often/always) for the following analyses. Based on the adjusted four‐point scale, item analysis was conducted. The results indicated strong psychometric properties. The corrected item‐total correlation coefficients ranged from 0.558 to 0.860 (> 0.40). Critical ratio tests demonstrated statistically significant differences between the high‐scoring and low‐scoring groups for all items (*t* > 3.00, *p* < 0.001), indicating good discriminative power. The overall Cronbach’s *α* for the initial item pool was 0.979. Furthermore, the analysis showed that deleting any single item did not significantly improve the Cronbach’s *α* coefficient; thus, all 37 items were retained for the subsequent factor analysis (see Supporting Table 3 for detailed item analysis results). Furthermore, multivariate normality was assessed using Mardia’s test. The results showed a multivariate kurtosis of 909.029 (*p* < 0.001), confirming that the data deviated from a multivariate normal distribution. Given the ordinal nature of the data and the non‐normal distribution, the polychoric correlation matrix was utilized for the subsequent EFA using FACTOR software to ensure accurate parameter estimation.

### 3.4. Validity Analysis Results

#### 3.4.1. Evidence Based on Test Content

The 37 items that comprised the final version of the Q‐NIMA had a I‐CVI above 0.78 (range 0.833–1.000). The S‐CVI of the entire questionnaire was 0.944 and was therefore considered excellent.

#### 3.4.2. Evidence Based on Internal Structure

##### 3.4.2.1. EFA

The suitability of the data for EFA was confirmed by the KMO test (KMO = 0.944) and Bartlett’s test of sphericity (*χ* ^2^ = 2634.5; df = 300; *p* < 0.001). Crucially, a retrospective analysis of the final factor solution demonstrated high average communalities (0.745) and strong factor loadings. As noted by Hogarty et al. [47], these specific data characteristics ensure that stable solutions can be obtained even with sample sizes significantly smaller than ours (*n* = 239), thereby validating the adequacy of the current sample size for robust analysis. EFA was conducted using FACTOR software (Version 12.06.07). Given the ordinal nature of the Likert‐scale data, polychoric correlations and the ULS extraction method were utilized, followed by Promin rotation to achieve factor simplicity. An iterative process was employed to refine the scale. Twelve items (items 16, 17, 24, 25, and 30–37) were sequentially removed due to insufficient factor loadings (< 0.40) or ambiguous cross‐loadings. After their removal, the total scale’s Cronbach’s *α* remained high (0.979 vs. 0.967).

The final EFA model retained 25 items distributed across five factors. While statistical criteria (e.g., PA) suggested a unidimensional structure, a five‐factor solution was selected to align with the theoretical framework and ensure interpretability. This structure explained 74.25% of the total variance. The five factors were labeled: nurse professional competence (NPC1–NPC3), nurse psychophysical state (NPS1–NPS6), patient‐related interruptions (PRI1‐PRI2), workplace colleague factors (WCF1–WCF4), and work system factors (WSF1–WSF10). As shown in Table 3, most items loaded distinctively on their respective factors. Three items displayed cross‐loadings (> 0.30): NPS4 (0.424 on NPC; 0.594 on NPS), WCF1 (0.376 on PRI; 0.507 on WCF), and WSF10 (0.429 on WCF; 0.827 on WSF). These items were retained and assigned to the factors where they exhibited the highest loadings and greatest theoretical interpretability.

**TABLE 3:** Exploratory factor analysis results of the Q‐NIMA (*n* = 239).

	Component					Communality (*h* ^2^)
	1 (NPC)	2 (NPS)	3 (PRI)	4 (WCF)	5 (WSF)	
NPC1	**0.801**	—	—	—	—	0.726
NPC2	**1.021**	—	—	—	—	0.918
NPC3	**0.542**	—	—	—	—	0.463
NPS1	—	**0.643**	—	—	—	0.680
NPS2	—	**0.653**	—	—	—	0.615
NPS3	—	**0.806**	—	—	—	0.736
NPS4	0.424	**0.594**	—	—	—	0.822
NPS5	—	**0.731**	—	—	—	0.840
NPS6	—	**0.767**	—	—	—	0.824
PRI1	—	—	**1.023**	—	—	0.910
PRI2	—	—	**0.698**	—	—	0.511
WCF1	—	—	0.376	**0.507**	—	0.674
WCF2	—	—	—	**0.986**	—	0.830
WCF3	—	—	—	**0.573**	—	0.504
WCF4	—	—	—	**0.830**	—	0.712
WSF1	—	—	—	0.344	**0.535**	0.855
WSF2	—	—	—	—	**0.365**	0.502
WSF3	—	—	—	—	**0.467**	0.773
WSF4	—	—	—	—	**0.733**	0.666
WSF5	—	—	—	—	**0.626**	0.808
WSF6	—	—	—	—	**0.788**	0.822
WSF7	—	—	—	—	**0.878**	0.844
WSF8	—	—	—	—	**0.831**	0.774
WSF9	—	—	—	—	**0.890**	0.876
WSF10	—	—	—	0.429	**0.827**	0.878
Eigenvalues	2.841	4.148	2.285	3.186	6.103	—
Variance percentage (%)	11.364	16.592	9.140	12.744	24.412	—
Cumulative (%)	11.364	27.956	37.096	49.840	74.252	—
Reliability (ORION)	0.948	0.948	0.938	0.933	0.970	—

*Note:* Response categories 4 and 5 were merged due to low endorsement rates. Extraction method: unweighted least squares (ULS). Rotation method: Promin. Loadings < 0.30 are suppressed. Bold values indicate the primary factor assignment.

Abbreviations: NPC, nurse professional competence; NPS, nurse psychophysical state; PRI, patient‐related interruptions; WCF, workplace colleague factors; WSF, work system factors.

##### 3.4.2.2. CFA

**FIGURE 1:**
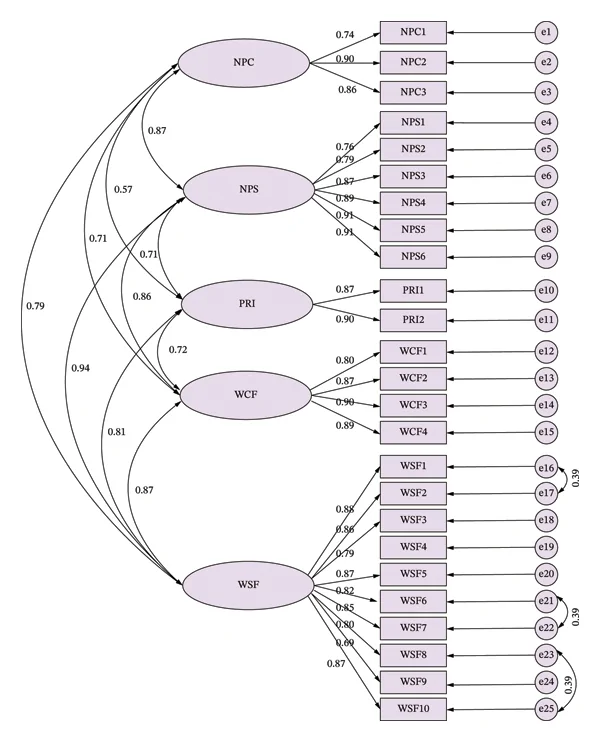
Standardized path coefficient diagram of the CFA model for the Q‐NIMA. Abbreviations: NPC: nurse professional competence; NPS: nurse psychophysical state; PRI: patient‐related interruptions; WCF: workplace colleague factors; WSF: work system factors.

Based on the EFA results, the first‐order five‐factor model was tested. To optimize model fit, we allowed for correlations between residual terms associated with high modification indices (see Figure 1). As shown in Table 1, the revised model exhibited satisfactory fit indices: *χ* ^2^/df = 2.455 (< 3), RMR = 0.028, and RMSEA = 0.078. Incremental fit indices (IFI, TLI, CFI) were 0.942, 0.933, and 0.942, respectively, all exceeding the 0.90 threshold. Parsimony indices were also satisfactory (PGFI = 0.660, PNFI = 0.791), confirming the robustness of the proposed structure.

To further validate the internal structure, we compared the proposed model with two alternatives (see Supporting Table 5). First, a one‐factor model (Model 1) was examined, where all items were loaded onto a single latent construct. This model exhibited a poor fit (*χ* ^2^/df = 4.672, CFI = 0.846, RMSEA = 0.125), indicating that the scale is not unidimensional and that a single factor cannot adequately explain the variance in the data. Second, a second‐order model (Model 3) was tested, assuming the five first‐order factors loaded onto a single higher‐order construct. While this model showed an acceptable fit (CFI = 0.931, RMSEA = 0.084), it did not fit the data as well as the proposed first‐order five‐factor model (Model 2). Specifically, Model 2 demonstrated the most favorable fit indices and the lowest AIC value (769.117) compared to the second‐order model (817.860). Consequently, the first‐order five‐factor model was retained as the optimal structure.

Convergent validity results are presented in Table 4. Standardized factor loadings ranged from 0.744 to 0.913 and were all statistically significant (*p* < 0.001). Ideally, composite reliability (CR) should exceed 0.7 and average variance extracted (AVE) should exceed 0.5; our results met these criteria, with CR ranging from 0.872 to 0.964 and AVE from 0.696 to 0.784. Regarding discriminant validity (Table 5), the square roots of the AVE were generally higher than the inter‐construct correlations. However, notable high correlations were found between NPS and WSF (0.939) and between WCF and WSF (0.872). These strong associations between the work system and nurses’ psychophysical states will be further interpreted in the Discussion section.

**TABLE 4:** Convergent validity of the Q‐NIMA.

Dimension	Items	Estimate	SE	CR value	AVE value
NPC	NPC1	0.744		0.872	0.696
	NPC2	0.897	0.092^∗∗∗^		
	NPC3	0.855	0.090^∗∗∗^		

NPS	NPS1	0.764		0.943	0.735
	NPS2	0.789	0.088^∗∗∗^		
	NPS3	0.869	0.087^∗∗∗^		
	NPS4	0.888	0.084^∗∗∗^		
	NPS5	0.913	0.084^∗∗∗^		
	NPS6	0.908	0.083^∗∗∗^		

PRI	PRI1	0.872		0.879	0.784
	PRI2	0.899	0.064^∗∗∗^		

WCF	WCF1	0.800		0.923	0.750
	WCF2	0.870	0.087^∗∗∗^		
	WCF3	0.898	0.081^∗∗∗^		
	WCF4	0.893	0.083^∗∗∗^		

WSF	WSF1	0.877		0.964	0.728
	WSF2	0.876	0.040^∗∗∗^		
	WSF3	0.883	0.052^∗∗∗^		
	WSF4	0.786	0.053^∗∗∗^		
	WSF5	0.873	0.050^∗∗∗^		
	WSF6	0.817	0.053^∗∗∗^		
	WSF7	0.848	0.051^∗∗∗^		
	WSF8	0.797	0.058^∗∗∗^		
	WSF9	0.895	0.050^∗∗∗^		
	WSF10	0.873	0.054^∗∗∗^		

*Note:* Response categories 4 and 5 were merged due to low endorsement rates.

Abbreviations: NPC, nurse professional competence; NPS, nurse psychophysical state; PRI, patient‐related interruptions; WCF, workplace colleague factors; WSF, work system factors.

^∗∗∗^< 0.001.

**TABLE 5:** Aggregated validity of the Q‐NIMA.

	NPC	NPS	PRI	WCF	WSF
NPC	**0.834**				
NPS	0.869^∗∗∗^	**0.857**			
PRI	0.570^∗∗∗^	0.711^∗∗∗^	**0.886**		
WCF	0.709^∗∗∗^	0.859^∗∗∗^	0.725^∗∗∗^	**0.866**	
WSF	0.792^∗∗∗^	0.939^∗∗∗^	0.810^∗∗∗^	0.872^∗∗∗^	**0.853**
AVE	0.696	0.735	0.784	0.75	0.728

*Note:* Bold diagonal values represent the square roots of the AVE.

Abbreviations: NPC, nurse professional competence; NPS, nurse psychophysical state; PRI, patient‐related interruptions; WCF, workplace colleague factors; WSF, work system factors.

^∗∗∗^< 0.001.

#### 3.4.3. Evidence Based on Relations to Other Variables

Spearman’s rank correlation coefficient revealed a significant positive association between the NIMA scale and the NASA‐TLX (*ρ* = 0.310, *p* < 0.001), indicating acceptable criterion‐related validity.

### 3.5. Reliability Analysis Results

The overall scale demonstrated excellent internal consistency, with a Cronbach’s *α* of 0.973. The Cronbach’s *α* coefficients for each dimension were as follows: NPC = 0.855, NPS = 0.928, PRI = 0.898, WCF = 0.899, and WSF = 0.960. The reliability of the retained factors (ORION) ranged from 0.933 to 0.970, indicating high precision in factor score estimation. Additionally, as presented in the CFA section, the CR values (0.872–0.964) further corroborated the scale’s internal consistency. The split‐half reliability of the total scale was 0.951, with Spearman–Brown coefficients of NPC = 0.848, NPS = 0.915, PRI = 0.898, WCF = 0.894, and WSF = 0.942. To assess test‐retest reliability, 30 participants were retested after a two‐week interval. The results indicated good stability, with an ICC (two‐way mixed model, absolute agreement) of 0.992 (95% CI = 0.984–0.996, *p* < 0.001).

## 4. Discussion

To our knowledge, this is the first questionnaire worldwide designed specifically to measure NIMA. Guided by the SEIPS model, we developed and validated a five‐domain, 25‐item self‐report instrument that reflects the work system. The domains are NPC, NPS, PRI, WCF, and WSF. Following COSMIN guidelines, we gathered validity evidence from multiple sources. The results support the structural validity, internal consistency, and stability of the Q‐NIMA, and it is suitable for routine clinical use. It moves beyond counting interruptions to identifying work‐system gaps that can be addressed through targeted interventions and monitored with clear process and outcome indicators.

### 4.1. The Questionnaire Demonstrates Innovative Value and Practical Significance

Nursing interruptions during medication administration occur frequently and often lead to adverse outcomes [8, 48]. Many existing studies use observation or generic self‐report tools, which do not describe risk and causes well in the medication context [18, 22, 25, 39, 48]. The Q‐NIMA measures five domains and helps us move from simply counting events to locating gaps in the work system that lead to interruptions. This tool adds three advances over existing self‐report scales. First, compared with the generic workplace interruption measure scale [25], it is anchored in key medication steps and uses nursing language, which improves relevance and interpretability at the bedside. Second, compared with the nursing interruption scales that focus mainly on people and environmental factors [22], it also covers tasks, tools and technology, and organizational factors, offering a fuller, action‐oriented view. Third, compared with the tool built for an emergency department [39], it is not confined to a single unit and can be used across wards and hospitals, supporting wider clinical applicability. These features enable risk profiling, root‐cause thinking, and targeted actions rather than simple event counting. In addition, the same framework can be adapted to other nursing activities, for example, transfusion or catheter care, by adjusting the items to those workflows.

### 4.2. The Questionnaire Is Developed With Methodological Rigor

This study was guided by the SEIPS [28] and by the risk evaluation index system for NIMA developed in our earlier work [31]. Based on the literature review and group discussions, we generated an initial item pool organized into five domains. We invited 12 experts for two rounds of expert inquiry to screen and revise the questionnaire items to determine the item pool. The authority coefficient of experts was > 0.900 and the Kendall harmony coefficient significance test was significant (*p* < 0.001), indicating good consistency of expert opinions and high recognition of the questionnaire items [39, 49]. To ensure relevance and usability, a presurvey was conducted with 30 nurses from diverse backgrounds. Through cognitive interviews, we observed and recorded nurses’ experiences while completing the questionnaire. Based on these insights, the questionnaire was simplified in terms of language and optimized in layout to enhance its applicability and improve data collection efficiency. Additionally, complex terminology was replaced with more accessible language to facilitate responses. We combined item analysis with rigorous psychometric testing to refine the questionnaire [50]. The Q‐NIMA demonstrated excellent content validity, with I‐CVIs ranging from 0.833 to 1.000 and an S‐CVI of 0.944 [51]. Notably, we utilized FACTOR software and polychoric correlation matrices for EFA, which is methodologically superior for ordinal Likert‐scale data. The EFA extracted five common factors explaining 74.25% of the total variance, with most item loadings exceeding 0.50, indicating strong structural validity. The communalities for the retained items were high (mean = 0.745, range: 0.463–0.918), and the KMO value was 0.944. According to methodological guidelines [38], these indicators confirm that our subsample size (*n* = 239) was sufficient to yield a stable factor solution, far exceeding the minimum requirements for data with high communalities. The CFA results confirmed that the model fit indices (e.g., *χ* ^2^/df, RMSEA, and RMR) met the recommended standards. Convergent validity was supported by high CR and AVE values. Although the high correlation between the NPS and WSF dimensions suggests a strong theoretical interplay between nurses’ internal states and their environment, the overall construct validity remains robust. In addition, criterion validity indicated the existence of a moderate correlation between the Q‐NIMA and the NASA‐TLX. The overall Cronbach’s *α* coefficient, split‐half reliability, and test‐retest reliability of the questionnaire were all above 0.8 [22, 39]. The results from the validity and reliability assessments confirm that the questionnaire possesses high scientific validity.

### 4.3. The Questionnaire Shows Clear Clinical Applicability

Guided by the SEIPS model, the five domains identified in this study mirror work‐system elements and indicate concrete levers for change. NPC represents the “person” element regarding technical proficiency, specifically focusing on the nurse’s familiarity with medication knowledge, equipment usage, and emergency response protocols. Distinct from competence, NPS captures the nurse’s internal physiological and psychological condition, including physical fatigue, emotional stability, self‐efficacy, and task readiness [1, 52]. This distinction allows managers to determine whether errors stem from a lack of skills (requiring training) or poor functional states (requiring workload adjustment or psychological support). Regarding social interactions, the questionnaire differentiates the sources of interruptions. PRI reflect demands from care recipients and their families, while WCF capture interruptions from the professional network, including physicians, managers, colleagues, and interns [49]. This separation is crucial for targeting interventions, such as implementing “do not disturb” vests for colleague interruptions versus educating families to reduce PRI. Finally, WSF serve as a comprehensive domain integrating environmental [31], technological [53], and organizational elements [31, 49]. It covers physical workspace issues (lighting, layout), resource availability (equipment, supplies), information integrity (medication orders, labels), and organizational management (staffing, scheduling, and team collaboration). By structuring scores along these specific dimensions, the Q‐NIMA moves beyond simple event counting. It identifies precisely where misalignments arise, whether in the nurse’s internal state, the social environment, or the broader work system, and how they propagate to medication interruptions.

In practice, nurse managers can administer the questionnaire at regular intervals (e.g., quarterly), compare domain scores across units, and monitor changes over time within the same unit. These comparisons highlight weak domains and translate directly into targeted management actions. For example, high scores in NPC prompt targeted microtraining on high‐alert medications, equipment operation workshops, and simulation drills for emergency scenarios. In contrast, high scores in NPS suggest the need for fatigue management strategies, reasonable scheduling to prevent burnout, and psychological support programs to enhance emotional stability and self‐efficacy [54, 55]. Regarding interruptions, high scores in PRI may require enhanced patient/family education regarding medication safety, whereas issues with WCF call for “sterile cockpit” protocols during medication rounds and improved interdisciplinary communication strategies [56]. Furthermore, high scores in WSF indicate systemic bottlenecks. Given the breadth of this domain, managers should analyze specific items to determine whether comprehensive interventions are needed in optimizing workspace layout and lighting [57], ensuring the availability of hardware and supplies, refining staffing ratios, or standardizing medication labels and order processing [58]. Operationally, triangulation with observations and device logs distinguishes what occurred and when from how it was appraised. Effectiveness can be tracked using process indicators such as interruption‐to‐resumption time and outcome indicators such as medication error rates, through before‐after analyses or statistical process control. Importantly, the system‐based structure of Q‐NIMA helps redirect attention from blaming individuals to optimizing organizational and environmental conditions, fostering a culture of shared responsibility, continuous monitoring, and reliable medication administration.

## 5. Limitations

This study has several limitations. First, the cross‐sectional design precludes causal inferences between the identified domains and medication safety outcomes. It also limits the assessment of the questionnaire’s stability over time. Second, reliance on self‐administered measures introduces common method variance and potential biases. Specifically, social desirability bias may lead nurses to underreport deficits in professional competence or psychophysical state. Furthermore, retrospective reporting may result in recall bias regarding interruption frequencies. Third, although the Q‐NIMA captures nurses’ perceived interruptions, which are crucial for understanding cognitive load, this study did not establish concurrent validity by correlating scores with objective metrics. Examples include observational logs or actual medication error rates. Future research should employ a mixed‐methods approach to triangulate subjective perceptions with objective data. Fourth, the use of convenience sampling via a web‐based platform may introduce selection bias. Nurses with excessive workloads, who are arguably most prone to interruptions, might have been underrepresented due to time constraints. Finally, the psychometric validation was conducted exclusively in a tertiary hospital in China. To ensure the tool’s robustness and generalizability, cross‐cultural validation and testing in diverse settings, such as primary care and community hospitals, are necessary.

## 6. Conclusions

This study developed a Q‐NIMA, following a standardized process for questionnaire development. The instrument comprises five dimensions and 25 items, demonstrating strong validity, reliability, scientific rigor, and practical applicability. It offers a multidimensional framework for evaluating NIMA, shedding light on its occurrence in China.

## 7. Implications for Nursing Management

By assessing NIMA events, this tool provides nursing managers with actionable insights to improve interruption management practices, minimize the adverse effects of interruptions, and enhance patient safety. This tool can serve as a critical resource for nursing managers to identify patterns of NIMA, guide the development and implementation of targeted intervention strategies, and optimize clinical workflows. By integrating the tool into daily nursing operations, managers can improve task management, allocate resources more effectively, and enhance overall nursing efficiency.

## Author Contributions

Xiaoqian Dong: writing–review and editing, writing–original draft, software, methodology, data curation, conceptualization, and funding acquisition; Nandan Chen and Xingxing Wang: software, methodology, data curation, and investigation; Cuilan Dong: methodology, data curation, and conceptualization; Li Yang: software, methodology, and data curation; Sha Wang: writing–review and editing, methodology, and investigation; Gang Gan and Min Liu: software, methodology, conceptualization, and investigation; Jianfei Xie: writing–review and editing, writing–original draft, methodology, investigation, conceptualization, funding acquisition, and project administration. The corresponding and first authors are the principal investigators.

## Funding

This work was supported by the Wisdom Accumulation and Talent Cultivation Project of the Third Xiangya Hospital of Central South University (No. BJ202205; PI: Jianfei Xie) and the Graduate Independent Exploration and Innovation Project of Central South University (No. 820; PI: Xiaoqian Dong).

## Disclosure

This manuscript follows the checklist items of the STROBE statement for cross‐sectional studies. The funders provided financial support only and had no role in the study design; data collection, analysis, or interpretation; manuscript writing; or the decision to submit the manuscript for publication. All authors have read and agreed to the published version of the manuscript.

## Ethics Statement

The Ethics Committee of Xiangya Nursing School of Central South University approved the study (No. E2022184). Participants provided informed consent before enrollment, with assurances of confidentiality upheld throughout the study. All collected data were handled with strict confidentiality.

## Conflicts of Interest

The authors declare no conflicts of interest.

## Supporting Information

Additional supporting information can be found online in the Supporting Information section.

## Supporting information


**Supporting Information 1** Supporting Table 1 presents the STROBE checklist for cross‐sectional studies.

**Supporting Information 2** Supporting Table 2 summarizes the expert panel consulted during scale development, including specialty, academic rank, and years of experience.

**Supporting Information 3** Supporting Table 3 reports item analysis results that support item screening and revision.

**Supporting Information 4** Supporting Table 4 details the normality test results (skewness and kurtosis) for the CFA subsample.

**Supporting Information 5** Supporting Table 5 provides a comparison of goodness‐of‐fit indices for the three alternative structural models tested.

## Data Availability

The data supporting the findings of this study are available from the corresponding author upon reasonable request.

## References

[1] Li J. , Wang S. , Wu X. et al., Factors to Predict the Knowledge, Attitude and Practice of Nursing Interruptions Among Nurses: A Nationwide Cross-Sectional Survey, Nurse Education in Practice. (2022) 64, 10.1016/j.nepr.2022.103428. 35970094

[2] Xie J. , Conceptual Analysis and Implications of Nursing Interruptions, Chinese Journal of Nursing. (2013) 48, no. 2, 175–178.

[3] Berg L. M. , Källberg A. S. , Göransson K. E. , Östergren J. , Florin J. , and Ehrenberg A. , Interruptions in Emergency Department Work: An Observational and Interview Study, BMJ Quality and Safety. (2013) 22, no. 8, 656–663, 10.1136/bmjqs-2013-001967. 23584208

[4] Zhao J. W. , Zhang X. , Lan Q. et al., Interruptions Experienced by Nurses During Pediatric Medication Administration in China: An Observational Study, Journal for Specialists in Pediatric Nursing. (2019) 24, no. 4, 10.1111/jspn.12265. 31332933

[5] Bonafide C. P. , Miller J. M. , Localio A. R. et al., Association Between Mobile Telephone Interruptions and Medication Administration Errors in a Pediatric Intensive Care Unit, JAMA Pediatrics. (2020) 174, no. 2, 162–169, 10.1001/jamapediatrics.2019.5001. 31860017 PMC6990809

[6] Alteren J. , Hermstad M. , Nerdal L. , and Jordan S. , Working in a Minefield; Nurses’ Strategies for Handling Medicine Administration Interruptions in Hospitals-A Qualtiative Interview Study, BMC Health Services Research. (2021) 21, no. 1, 10.1186/s12913-021-07122-8. PMC8518177 34649559

[7] Ma K. , Research Progress on Nursing Interruptions During Medication Administration and Management Strategies, Journal of Nursing Science. (2018) 33, no. 18, 21–24.

[8] Wang W. , Current Status and Influencing Factors of Nursing Interruption Events, American Journal of Managed Care. (2021) 27, no. 6, E188–E194. 34156222 10.37765/ajmc.2021.88667

[9] Danesh V. , Sasangohar F. , Kallberg A. S. , Kean E. B. , Brixey J. J. , and Johnson K. D. , Systematic Review of Interruptions in the Emergency Department Work Environment, International Emergency Nursing. (2022) 63, 10.1016/j.ienj.2022.101175. 35843150

[10] Wudu M. A. , Bayked E. M. , Bekalu Y. E. , and Birhanu T. A. , Prevalence of Medication Administration Errors and Its Determinants Among Nurses in Ethiopia: A Systematic Review and Meta-Analysis, BMC Nursing. (2025) 24, no. 1, 10.1186/s12912-025-03186-7. PMC12083152 40380296

[11] Schroers G. , Tell D. , and O’Rourke J. , Association of External Interruptions With Increased Medication Administration Duration and Self-Interruptions: A Direct Observational Study: Empirical Research Quantitative, Journal of Advanced Nursing. (2023) 79, no. 11, 4339–4347, 10.1111/jan.15674. 37070669

[12] Liu Q. Q. , Workflow Interruptions, Perceived Workload and Missed Nursing: Their Impact on Nurses’ Health Status-A Structural Equation Model, Journal of Advanced Nursing. (2025) . 10.1111/jan.16855 39991959

[13] Teigné D. , Cazet L. , Birgand G. et al., Improving Care Safety by Characterizing Task Interruptions During Interactions Between Healthcare Professionals: An Observational Study, International Journal for Quality in Health Care. (2023) 35, no. 3, 10.1093/intqhc/mzad069. PMC10507660 37688401

[14] Zoupanou Z. and Rydstedt L. W. , The Mediating and Moderating Role of Affective Rumination Between Work Interruptions and Well-Being, Work. (2019) 62, no. 4, 553–561, 10.3233/wor-192890. 31104041

[15] Körner B. , Debus M. E. , Wu C. , and Kleinmann M. , How and When Do Frequent Daily Work Interruptions Contribute to or Undermine Daily Job Satisfaction? A Stress Appraisal Perspective, Journal of Organizational Behavior. (2025) 46, no. 1, 1–23, 10.1002/job.2833.

[16] Shan Y. , Shang J. , Yan Y. , and Ye X. , Workflow Interruption and Nurses’ Mental Workload in Electronic Health Record Tasks: an Observational Study, BMC Nursing. (2023) 22, no. 1, 10.1186/s12912-023-01209-9. PMC9996908 36890555

[17] Freitas W. C. J. D. , Distractions and Interruptions in Medication Preparation and Administration in Inpatient Units, 2020.

[18] Lin T. , Feng X. , Gao Y. et al., Nursing Interruptions in Emergency Room in China: An Observational Study, Journal of Nursing Management. (2021) 29, no. 7, 2189–2198, 10.1111/jonm.13372. 33993569

[19] Forsyth K. L. , Hawthorne H. J. , El-Sherif N. et al., Interruptions Experienced by Emergency Nurses: Implications for Subjective and Objective Measures of Workload, Journal of Emergency Nursing. (2018) 44, no. 6, 614–623, 10.1016/j.jen.2018.02.001. 29655927

[20] Healey A. N. , Sevdalis N. , and Vincent C. A. , Measuring Intra-Operative Interference From Distraction and Interruption Observed in the Operating Theatre, Ergonomics. (2006) 49, no. 5-6, 589–604, 10.1080/00140130600568899. 16717011

[21] Gorman G. E. , Qualitative Research for the Information Professional: A Practical Handbook, 2005, Facet.

[22] Yu E. and Lee E. , Development and Validation of a Nursing Work Interruption Scale, International Journal of Environmental Research and Public Health. (2022) 19, no. 20, 10.3390/ijerph192013487. PMC9602459 36294067

[23] Grundgeiger T. , Dekker S. , Sanderson P. , Brecknell B. , Liu D. , and Aitken L. M. , Obstacles to Research on the Effects of Interruptions in Healthcare, BMJ Quality and Safety. (2016) 25, no. 6, 392–395, 10.1136/bmjqs-2015-004083. 26658346

[24] Lin B. C. , Kain J. M. , and Fritz C. , Don’t Interrupt me! an Examination of the Relationship Between Intrusions at Work and Employee Strain, Educational Publishing Foundation: US. (2013) 20, no. 2, 77–94, 10.1037/a0031637.

[25] Wilkes S. M. , Barber L. K. , and Rogers A. P. , Development and Validation of the Workplace Interruptions Measure, Stress and Health. (2018) 34, no. 1, 102–114, 10.1002/smi.2765. 28639737

[26] Jalali A. , Babaei K. , Sharifi A. et al., Psychometric Properties of the Persian Version of Nursing Work Interruption Scale, BMC Nursing. (2025) 24, no. 1, 10.1186/s12912-025-02837-z. PMC11837290 39972284

[27] Chen L. et al., Sinicisation and Reliability Testing of the Nursing Work Interruption Scale, Nursing and Rehabilitation. (2024) 23, no. 4, p12–p15.

[28] Carayon P. , Wooldridge A. , Hoonakker P. , Hundt A. S. , and Kelly M. M. , SEIPS 3.0: Human-Centered Design of the Patient Journey for Patient Safety, Applied Ergonomics. (2020) 84, 10.1016/j.apergo.2019.103033. PMC7152782 31987516

[29] Schutijser B. , Klopotowska J. E. , Jongerden I. P. , Spreeuwenberg P. M. M. , De Bruijne M. C. , and Wagner C. , Interruptions During Intravenous Medication Administration: A Multicentre Observational Study, Journal of Advanced Nursing. (2019) 75, no. 3, 555–562, 10.1111/jan.13880. 30334590

[30] Wang X. , The Occurrence of ICU Nursing Interruptions and Their Relationship With the Work Stress of ICU Nurses, International Journal of Nursing. (2023) 42, no. 5, p814–p818.

[31] Dong X. , Construction and Application of Risk Evaluation Index System of Nursing Interruptions in Medication Process, Chinese Journal of Emergency and Critical Care Nursing. (2024) 5, no. 08, 731–736.

[32] Alsaadi D. , Tierney A. , McErlean K. , Moran L. , Kavanagh O. , and McMonagle M. , Introduction of a Bleepless Intern on-Call Era, The Surgeon. (2024) 22, no. 3, 154–157, 10.1016/j.surge.2024.02.004. 38485634

[33] Huang X. , A Survey on Nursing Interruption During Medication Administration Process in Respiratory Department, Chinese Journal of Nursing. (2015) 50, no. 12, 1489–1493.

[34] Who , State of the World’s Nursing 2020: Investing in Education, Jobs and Leadership, 2020.

[35] Cai Y. , Li Q. , Cao T. , and Wan Q. , Nurses’ Work Engagement: the Influences of Ambidextrous Leadership, Clinical Nurse Leadership and Workload, Journal of Advanced Nursing. (2023) 79, no. 3, 1152–1161, 10.1111/jan.15086. 34723406

[36] MacCallum R. C. , Widaman K. F. , Preacher K. J. , and Hong S. , Sample Size in Factor Analysis: The Role of Model Error, Multivariate Behavioral Research. (2001) 36, no. 4, 611–637, 10.1207/s15327906mbr3604_06. 26822184

[37] Ferrando P. J. , Lorenzo-Seva U. , Hernández-Dorado A. , and Muñiz J. , Decalogue for the Factor Analysis of Test Items, Psicothema. (2022) 34, no. 1, 7–17, 10.7334/psicothema2021.456. 35048890

[38] Lloret-Segura S. , Exploratory Item Factor Analysis: A Practical Guide Revised and Up-Dated, Anales de Psicología/Annals of Psychology. (2014) 30, no. 3, 1151–1169.

[39] Lin T. , Feng X. , and Gao Y. , Development and Evaluation of Psychometric Properties of a Chinese Version Questionnaire for Measuring Emergency Nursing Interruptions, Journal of Nursing Management. (2024) 2024, no. 1, 10.1155/2024/8750135. PMC11919178 40224780

[40] Polit D. F. and Beck C. T. , Nursing Research: Generating and Assessing Evidence for Nursing Practice, 2021, Wolters Kluwer.

[41] Wu M. L. , Questionnaire Statistical Analysis Practice: SPSS Operation and Application, 2010, Chongqing University Press.

[42] Hu L. and Bentler P. M. , Cutoff Criteria for Fit Indexes in Covariance Structure Analysis: Conventional Criteria Versus New Alternatives, Structural Equation Modeling. (1999) 6, no. 1, 1–55, 10.1080/10705519909540118.

[43] Mulaik S. A. , James L. R. , Van Alstine J. et al., Evaluation of Goodness-of-Fit Indices for Structural Equation Models, 1989, American Psychological Association: US, 430–445.

[44] Laing L. , Chinesization, Reliability and Validity Test of National Aeronautics and Space Administration Taskload Index, Chinese Nursing Research. (2019) 33, no. 05, 734–737.

[45] Doval E. , Viladrich C. , and Angulo-Brunet A. , Coefficient Alpha: The Resistance of a Classic, Psicothema. (2023) 35, no. 1, 5–20, 10.7334/psicothema2022.321. 36695846

[46] Ferrando P. J. and Lorenzo-Seva U. , A Note on Improving EAP Trait Estimation in Oblique Factor-Analytic and Item Response Theory Models, 2016, Universitat de València, 235–247.

[47] Hogarty K. Y. , Hines C. V. , Kromrey J. D. , Ferron J. M. , and Mumford K. R. , The Quality of Factor Solutions in Exploratory Factor Analysis: The Influence of Sample Size, Communality, and Overdetermination, Educational and Psychological Measurement. (2005) 65, no. 2, 202–226, 10.1177/0013164404267287.

[48] Eid T. , Machudo S. , and Eid R. , Interruptions During Medication Work in a Saudi Arabian Hospital: An Observational and Interview Study of Nurses, Journal of Nursing Scholarship. (2022) 54, no. 5, 639–647, 10.1111/jnu.12765. 35064618

[49] Xie J. , Sun Q. , Tang S. et al., Knowledge, Attitude and Practice Regarding Nursing Interruptions Among Chinese Nurses: A Nationwide Cross-Sectional Survey, International Journal of Nursing Science. (2020) 7, no. 1, 66–73, 10.1016/j.ijnss.2019.12.004. PMC7031111 32099862

[50] Wang X. , Zhang C. , Qi Y. et al., Digital Health Literacy Questionnaire for Older Adults: Instrument Development and Validation Study, Journal of Medical Internet Research. (2025) 27, 10.2196/64193. PMC11966078 40106815

[51] Shi J. , Mo X. , and Sun Z. , [Content Validity Index in Scale Development], Zhong Nan Da Xue Xue Bao Yi Xue Ban. (2012) 37, no. 2, 152–155. 22561427 10.3969/j.issn.1672-7347.2012.02.007

[52] Shao G. T. , Tian S. , Tian F. , Chen L. , and Zhao Y. , The Impact of Task Interruptions on the Unsafe Behavior of Coal Mine Tunneling Machine Operators: the Moderating Role of Fatigue, Applied Sciences-Basel. (2025) 15, no. 5, 10.3390/app15052764.

[53] Ma J. , Bai Y. , Xie D. S. , and Yang G. F. , Factors Influencing the Interruption of Nursing Document Writing in the Intensive Care Unit: A Cross-Sectional Survey, Journal of Multidisciplinary Healthcare. (2023) 16, 419–427, 10.2147/jmdh.s394817. 36820218 PMC9938661

[54] Vital C. J. , Nathanson B. H. , Effects of the Interruption Management Strategy , and Stay S. A. F. E. , During Medication Administration, Rehabilitation Nursing. (2023) 48, no. 2, 65–74. 36792960 10.1097/RNJ.0000000000000404

[55] Owen S. , Menzies J. , and Pontefract S. , Educational Interventions to Reduce Nurse Medication Interruptions: A Scoping Review, Nurse Education Today. (2023) 121, 10.1016/j.nedt.2022.105665. 36527755

[56] Yoder M. , Schadewald D. , and Dietrich K. , The Effect of a Safe Zone on Nurse Interruptions, Distractions, and Medication Administration Errors, Journal of Infusion Nursing. (2015) 38, no. 2, 140–151, 10.1097/nan.0000000000000095. 25723837

[57] Sloane J. F. , Donkin C. , Newell B. R. , Singh H. , and Meyer A. N. D. , Managing Interruptions to Improve Diagnostic Decision-Making: Strategies and Recommended Research Agenda, Journal of General Internal Medicine. (2023) 38, no. 6, 1526–1531, 10.1007/s11606-022-08019-w. 36697925 PMC10160308

[58] Rosenblatt S. A. , Balmer D. F. , and Boyer D. L. , Creating a “Culture of Triage”: A Dual-Perspective Study of Interruptions During ICU Rounds, Pediatric Critical Care Medicine. (2021) 22, no. 2, 172–180, 10.1097/pcc.0000000000002595. 33065734

