# Supplementary material for: Perceived Nursing Interruptions During Medication Administration: Development and Validation of a Questionnaire Among Chinese Nurses
Source: J Nurs Manag. 2026 Sep 7;2026:5881570. doi: 10.1155/jonm/5881570 (PMC13548313; doi:10.1155/jonm/5881570)
Supplement: Supplementary file 2 [file JONM-2026-5881570-s002.docx]

| **Supplementary Table 2 Experts Personal Information** | | | | | | | | | |
| --- | --- | --- | --- | --- | --- | --- | --- | --- | --- |
| No. | **Gender** | **Age（year）** | **Length of service（year）** | **Educational background** | **Professional title** | **Work Unit** | **Supervisor qualification** | **Safety management experience** | **Research direction** |
| 1 | Female | 43 | 17 | Doctor | Professor | The Second Xiangya Hospital of Central South University | Master's Supervisor | Yes | Nursing management and nursing psychology |
| 2 | Male | 38 | 10 | Master | Associate Professor | The Third Xiangya Hospital of Central South University | NO | NO | Medical management |
| 3 | Female | 35 | 5 | Doctor | Lecturer | Hunan University of Chinese Medicine | NO | NO | Nursing management and nursing psychology |
| 4 | Female | 44 | 17 | Master | Professor | The Dongguan Hospital of Guangdong Province | Master's Supervisor | Yes | Nursing management and nursing psychology |
| 5 | Male | 42 | 17 | Doctor | Associate Professor | The Third Xiangya Hospital of Central South University | NO | NO | Medical management |
| 6 | Female | 32 | 4 | Doctor | Associate Professor | The Xiangya Nursing School of Central South University | Master's Supervisor | NO | Nursing psychology |
| 7 | Female | 31 | 5 | Master | nurse-in-charge | The First Hospital of Zhengzhou University | NO | NO | Clinical nursing |
| 8 | Female | 63 | 44 | Bachelor | Professor | Aier Eye Hospital (Shan Xi) | Master's Supervisor | Yes | Nursing management and nursing psychology |
| 9 | Male | 33 | 8 | Doctor | Professor | Central South University | Doctoral Supervisor | Yes | Nursing management |
| 10 | Female | 34 | 9 | Master | Associate Professor | The Third Xiangya Hospital of Central South University | NO | Yes | Nursing management |
| 11 | Male | 61 | 38 | Master | Professor | The First Hospital of Chongqing Medical University | Doctoral Supervisor | Yes | Medical management |
| 12 | Female | 52 | 33 | Doctor | Professor | The Third Xiangya Hospital of Central South University | Master's Supervisor | Yes | Nursing management and nursing psychology |
