# Supplementary material for: Perceived Nursing Interruptions During Medication Administration: Development and Validation of a Questionnaire Among Chinese Nurses
Source: J Nurs Manag. 2026 Sep 7;2026:5881570. doi: 10.1155/jonm/5881570 (PMC13548313; doi:10.1155/jonm/5881570)
Supplement: Supplementary file 3 [file JONM-2026-5881570-s003.docx]

**Supplementary Table 3 Item analysis results (n=239)**

| **Item** | **Critical ratio** | | **Corrected Item-Total Correlation** | Cronbach's alpha coefficient（0.979） | **Delete or not** |
| --- | --- | --- | --- | --- | --- |
|  | t | p |  |  |  |
| X1 | 10.367 | 0 | 0.584 | 0.979 | No |
| X2 | 8.757 | 0 | 0.629 | 0.979 | No |
| X3 | 10.981 | 0 | 0.698 | 0.979 | No |
| X4 | 14.328 | 0 | 0.675 | 0.979 | No |
| X5 | 9.935 | 0 | 0.649 | 0.979 | No |
| X6 | 12.808 | 0 | 0.704 | 0.979 | No |
| X7 | 11.057 | 0 | 0.747 | 0.979 | No |
| X8 | 10.930 | 0 | 0.760 | 0.979 | No |
| X9 | 15.854 | 0 | 0.759 | 0.979 | No |
| X10 | 12.279 | 0 | 0.593 | 0.979 | No |
| X11 | 11.863 | 0 | 0.640 | 0.979 | No |
| X12 | 12.811 | 0 | 0.651 | 0.979 | No |
| X13 | 8.965 | 0 | 0.649 | 0.979 | No |
| X14 | 13.150 | 0 | 0.758 | 0.979 | No |
| X15 | 8.614 | 0 | 0.660 | 0.979 | No |
| X16 | 8.445 | 0 | 0.696 | 0.979 | No |
| X17 | 12.199 | 0 | 0.795 | 0.978 | No |
| X18 | 13.676 | 0 | 0.829 | 0.978 | No |
| X19 | 16.814 | 0 | 0.823 | 0.978 | No |
| X20 | 18.616 | 0 | 0.815 | 0.978 | No |
| X21 | 15.756 | 0 | 0.669 | 0.979 | No |
| X22 | 15.968 | 0 | 0.837 | 0.978 | No |
| X23 | 18.470 | 0 | 0.778 | 0.978 | No |
| X24 | 16.874 | 0 | 0.703 | 0.979 | No |
| X25 | 18.143 | 0 | 0.725 | 0.979 | No |
| X26 | 17.982 | 0 | 0.786 | 0.978 | No |
| X27 | 17.333 | 0 | 0.777 | 0.978 | No |
| X28 | 17.503 | 0 | 0.839 | 0.978 | No |
| X29 | 14.507 | 0 | 0.822 | 0.978 | No |
| X30 | 14.467 | 0 | 0.842 | 0.978 | No |
| X31 | 14.716 | 0 | 0.855 | 0.978 | No |
| X32 | 13.270 | 0 | 0.840 | 0.978 | No |
| X33 | 12.410 | 0 | 0.837 | 0.978 | No |
| X34 | 13.905 | 0 | 0.856 | 0.978 | No |
| X35 | 13.587 | 0 | 0.860 | 0.978 | No |
| X36 | 14.673 | 0 | 0.558 | 0.979 | No |
| X37 | 14.419 | 0 | 0.777 | 0.978 | No |
