# Supplementary material for: Perceived Nursing Interruptions During Medication Administration: Development and Validation of a Questionnaire Among Chinese Nurses
Source: J Nurs Manag. 2026 Sep 7;2026:5881570. doi: 10.1155/jonm/5881570 (PMC13548313; doi:10.1155/jonm/5881570)
Supplement: Supplementary file 4 [file JONM-2026-5881570-s004.docx]

| **Supplementary Table 4** Normality Test for CFA Subsample (n=239) | | | | | |
| --- | --- | --- | --- | --- | --- |
| **Item** | **N** | **Skewness Values** | | **Kurtosis Values** | |
|  |  | **Statistic** | **Standard Error** | **Statistic** | **Standard Error** |
| NPC1 | 239 | 0.291 | 0.157 | -0.627 | 0.314 |
| NPC2 | 239 | 0.847 | 0.157 | -0.53 | 0.314 |
| NPC3 | 239 | 0.873 | 0.157 | -0.481 | 0.314 |
| NPS1 | 239 | 0.267 | 0.157 | -0.451 | 0.314 |
| NPS2 | 239 | 0.749 | 0.157 | -0.504 | 0.314 |
| NPS3 | 239 | 0.614 | 0.157 | -0.742 | 0.314 |
| NPS4 | 239 | 0.784 | 0.157 | -0.506 | 0.314 |
| NPS5 | 239 | 0.866 | 0.157 | -0.373 | 0.314 |
| NPS6 | 239 | 0.719 | 0.157 | -0.616 | 0.314 |
| PRI1 | 239 | 0.253 | 0.157 | -0.681 | 0.314 |
| PRI2 | 239 | 0.339 | 0.157 | -0.761 | 0.314 |
| WCF1 | 239 | 0.416 | 0.157 | 0.232 | 0.314 |
| WCF2 | 239 | 0.533 | 0.157 | -0.756 | 0.314 |
| WCF3 | 239 | 0.394 | 0.157 | -0.633 | 0.314 |
| WCF4 | 239 | 0.577 | 0.157 | -0.376 | 0.314 |
| WSF1 | 239 | 0.952 | 0.157 | -0.165 | 0.314 |
| WSF2 | 239 | 0.661 | 0.157 | -0.605 | 0.314 |
| WSF3 | 239 | 0.620 | 0.157 | -0.571 | 0.314 |
| WSF4 | 239 | 0.369 | 0.157 | -0.356 | 0.314 |
| WSF5 | 239 | 0.705 | 0.157 | -0.23 | 0.314 |
| WSF6 | 239 | 0.407 | 0.157 | -0.47 | 0.314 |
| WSF7 | 239 | 0.441 | 0.157 | -0.512 | 0.314 |
| WSF8 | 239 | 0.462 | 0.157 | -0.751 | 0.314 |
| WSF9 | 239 | 0.756 | 0.157 | -0.324 | 0.314 |
| WSF10 | 239 | 0.817 | 0.157 | -0.541 | 0.314 |
