# Supplementary material for: Perceived Nursing Interruptions During Medication Administration: Development and Validation of a Questionnaire Among Chinese Nurses
Source: J Nurs Manag. 2026 Sep 7;2026:5881570. doi: 10.1155/jonm/5881570 (PMC13548313; doi:10.1155/jonm/5881570)
Supplement: Supplementary file 5 [file JONM-2026-5881570-s005.docx]

**Supplementary Table 5** Comparison of Fit Indices for Alternative Models

| **Model** | **X2/df** | **RMSEA** | **IFI** | **TLI** | **CFI** | **Comparison** | **AIC** |
| --- | --- | --- | --- | --- | --- | --- | --- |
|  | ＜3 | ＜0.08 | ＞0.9 | ＞0.9 | ＞0.9 |  |  |
| Model 1: One-Factor Model | 4.672 | 0.125 | 0.846 | 0.832 | 0.846 | Reference | 1384.899 |
| Model 2: First-Order Five-Factor Model | 2.455 | 0.078 | 0.942 | 0.933 | 0.942 | vs. Model 1 | 769.117 |
| Model 3: Second-Order Model | 2.689 | 0.084 | 0.932 | 0.923 | 0.931 | vs. Model 2 | 817.860 |

Note. RMSEA = Root Mean Square Error of Approximation; IFI = Incremental Fit Index; TLI = Tucker-Lewis Index; CFI = Comparative Fit Index; AIC = Akaike Information Criterion. Model 1: All items loading on a single factor. Model 2: Items loading on their respective five theoretical factors. Model 3: Five first-order factors loading on a single second-order factor.
